# Environmental Kuznets Curve Analysis of the Economic Development and Nonpoint Source Pollution in the Ningxia Yellow River Irrigation Districts in China

**DOI:** 10.1155/2013/267968

**Published:** 2013-09-21

**Authors:** Chunlan Mao, Ningning Zhai, Jingchao Yang, Yongzhong Feng, Yanchun Cao, Xinhui Han, Guangxin Ren, Gaihe Yang, Qing-xiang Meng

**Affiliations:** ^1^College of Forestry, Northwest A&F University, Yangling, Shaanxi 712100, China; ^2^The Research Center of Recycle Agricultural Engineering and Technology of Shaanxi Province, Yangling, Shaanxi 712100, China; ^3^College of Agronomy, Northwest A&F University, Yangling, Shaanxi 712100, China; ^4^College of Resources and Environment, Henan Agricultural University, Zhengzhou, Henan 450002, China

## Abstract

This study applies the environmental Kuznets curve to test the relationship between the regional economic growth and the different types of agricultural nonpoint source pollution loads in the Ningxia Yellow River irrigation area by using the Johnes export coefficient method. Results show that the pollution load generated by crop cultivation and livestock-breeding industries in the Ningxia Yellow River irrigation area shows an inverted U-shaped feature; however, this feature is absent in living-sewage pollution load. Crop pollution has shown a decreasing trend since 1997 because of the increased per capita income of farmers. Livestock-breeding pollution load reached its turning point when the per capita income of farmers reached 8386.74 RMB. Therefore, an increase in the per capita income of farmers corresponds to an increase in the livestock-breeding pollution load in the Ningxia Yellow River irrigation area.

## 1. Introduction

Agricultural nonpoint source pollution has become a growing environment problem, contributing so much to water eutrophication in the world [1] and influencing 30% to 50% of the total land area of the world. In addition, 12% of 1200 million ha of degraded land is caused by agricultural nonpoint source pollution [2, 3]. According to the Environmental Protection Agency, agricultural nonpoint source pollution accounts for 52% of the total nitrogen (TN) in surface waters and covers 60% to 87% of the basin total input in Sweden, 60% of environment load in the Netherlands, and 94% of 270 rivers in Denmark [4]. Also, nonpoint source pollution represents more than 94% of nutrient loads except for mineral phosphorus (50%) [5]. Overall, the loss of nitrogen and phosphorus can be transported by farmland drainage and surface water to water which results in deterioration of water quality and accelerates agricultural nonpoint source pollution. According to the survey, 80% of 532 rivers all suffered from nitrogen pollution [6]. Every year, approximately 92% and 88% of the TN in the Yangtze River and Yellow River, respectively, came from agriculture sources, in which half of these values were caused by fertilizers [7].

Environmental pollution has become a serious concern because of rapid industrialization and resource depletion [8]. In 1955, Kuznets found that the relationship between income inequality and economic growth has an inverted U-shaped feature [9]. This relationship is denoted as the environmental Kuznets curve (EKC). EKC implies that in the early stages of economic development, the environment paid a high price for economic growth because people used technology to exploit resources [10–12]. In other words, environmental quality degradation increases in early stage of economic growth and slows down in later stage as economy develops. Thus, the EKC reveals a dynamic changeable process of environmental quality as the fortunes [13]. One reason an EKC might emerge is, namely, the absence of corresponding willingness for reducing environmental damages with initial income growth [14]. Many literatures have reviewed theoretical developments, empirical studies and contributions of EKC, and challenges, analyzed indicators of environmental degradation favoring the EKC hypothesis, and proposed that developing countries could benefit from developed countries standards [13, 15, 16]. Copeland and Taylor [17] noted that the EKC literature demonstrated the important potential for an income effect on environment quality focusing on the relation between economic growth and environmental degradation.

Economic growth as well as science and technology advancements has fostered public awareness regarding environment protection; the application of advanced environmental management techniques results in the gradual reduction of harmful emissions, thereby substantially improving environmental condition [18–20]. Since its proposal, the EKC hypothesis has been widely used in developed countries. However, not all studies fit the EKC hypothesis. In 1991, Grossman and Krueger analyzed urban air quality by using the global environmental monitoring system and found that the relationship between SO_2_ and smoke fits the inverted EKC hypothesis; that is, the content of suspended particles in the atmosphere increases when the per capita GDP has vertex ranging from $4000 to $5000 [21]. Currently, several investigations on EKC have already been performed in China. Li and Bao [22] analyzed the trend changes in the “three wastes” (gas waste, water waste, and residues waste) in eastern, western, and central China. Their results showed that these areas have not reached the EKC turning point for pollutants. The indices in Beijing [23] and Shanghai [24] have exceeded this turning point and showed decreasing trends. In Anhui province, the EKC showed U-shaped and inverted U-shaped features [25]. EKC is mostly associated with industrial pollution and is rarely applied in analyzing the relationship between agricultural pollution and economics.

The Ningxia Yellow River irrigation area not only supports the social economic development of Ningxia but also halts the Tengger and Mu Us Deserts from spreading. The Ningxia Yellow River irrigation area is more affected by ecological factors than by economic factors. However, large amounts of returned water considerably influence the water environment in the Yellow River downstream. Currently, limited research is available on the relationship between agricultural economic growth and ecological environment changes. The statistical data of 11 counties in the Ningxia Yellow River irrigation area were collected, arranged, and analyzed. The characteristics of animal manure and rural domestic pollution (i.e., human feces and rural domestic sewage) were estimated and evaluated according to national standards and relevant literature. The EKC hypothesis was applied to find a curve that fits the data on animal manure and rural domestic pollution. This study has a theoretical and realistic significance in the control of agricultural nonpoint source pollution and in the environmental maintenance in the Yellow River irrigation area.

## 2. Materials and Methods

### 2.1. Experimental Area

**Figure 1:**
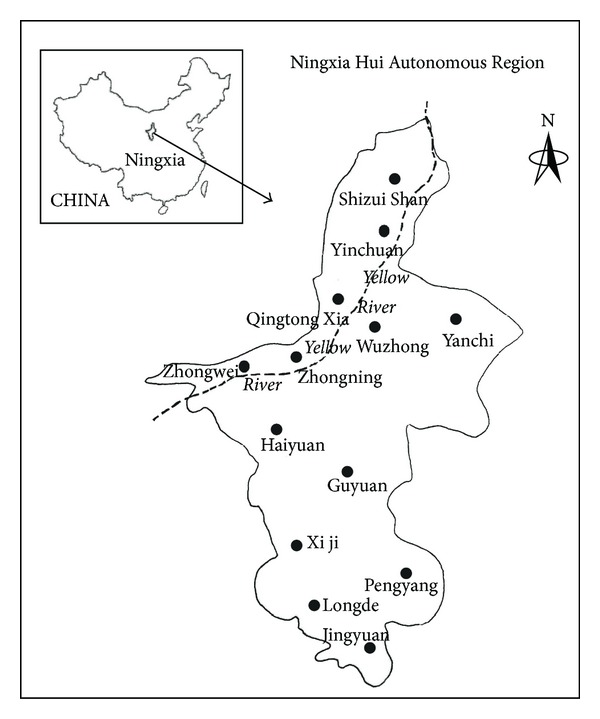
Location of the study area at the Yellow River irrigation area in the northern part of Ningxia along the Yellow River, China.

The Ningxia Yellow River irrigation area is divided into Qingtong Xia and Weining irrigation districts, involving Yinchuan City, Zhongwei City (except HaiRMB county), Wuzhong City (except Yanchi and Tongxin counties), and Shizuishan City (see Figure 1). A total of 11 counties and 20 state-owned farms and animal farms were included. The agricultural population is 1678 thousand, covering 48.4% of the total population (i.e., 3468 thousand). The Ningxia Yellow River irrigation area is a typical area of rice and corn crop rotation in the northwest arid and semiarid area in China and receives annually an average of 7 billion m^3^ of water from the Yellow River, approximately 93% to 95% of which is used as agricultural water and only 25 billion m^3^ of water returns to the Yellow River. On the basis of the monitoring reports, the water quality of the main ditches of the Yellow River irrigation districts is of inferior class V and has high NO^3−^–N and NH^4+^–N contents [26]. Approximately 61% to 66% TN and 76% to 81% NH^4+^–N came from farming. The NH^4+^–N concentration in drainage was generally 20 mg/L to 30 mg/L and even reaches 70 mg/L, thus inducing substantial influence on the water quality along the Lower Yellow River [27]. From 2002 to 2007, the average amount of N fertilizer applied was 301 kg/hm^2^, which is 1.6 times the national average amount [28].

### 2.2. Experimental Methods

#### 2.2.1. Estimation Method of Nonpoint Source Pollution Load

Nonpoint source pollution load must first be estimated before estimating the losses of nonpoint source pollution in these districts. This study adopted the Jones export coefficient method [29], which directly establishes the relationship between land use and agricultural nonpoint pollution load of receiving water using easily accessible information such as land utilization conditions. The Jones export coefficient method is expressed as follows:

(1) $$\begin{array}{c} L=\overset{n}{\underset{i=1}{\sum }}E_{i}\left[A_{i}\left(I_{i}\right)\right]+P, \end{array}$$

where *L* is the total pollution (pollutant load) in kg; *E* is the output coefficient of type *i* pollutants in kg · km^−2·a−1^, which is the year output of unit area per capita or pollution of each head of livestock; *A*_*i*_ is the land use area of type *i* livestock, which is in km^2^; type *i* pollutant is in kg; *P* is the amount of pollutant with rainfall, which is ignored in this study as the EKC relationships are more likely to hold for pollutants of more short-term and local impacts with, rather than those with more global, indirect, and long-term impacts.

#### 2.2.2. Selection of the EKC Model

The general model of EKC is as follows (see [30]):

(2) $$\begin{array}{c} E=\alpha +\beta_{1}Y+\beta_{2}Y_{2}+\beta_{3}Y_{3}+u, \end{array}$$

where *E* is the pollution index of a certain county or area; *Y* is the economic growth indicator usually replaced by per capita GDP (this study utilized the per capita net income of farmers); *α* is the intercept; *β*_1_, *β*_2_, and *β*_3_ are unknown parameters; *u* is a random error. The turning point of the model can be obtained through first curvature vector. The “turning point” is the point beyond which increases in economic development result in reduction in environmental pollution, as expressed in the following equation:

(3) $$\begin{array}{c} X_{t}=\frac{\partial Y}{\partial X}. \end{array}$$

The following relationships were observed between agricultural nonpoint source pollution and economic development levels which can be categorized into 7 types including: (1) monotonic increasing, (2) monotonic decreasing, (3) inverted U-shaped (EKC type, where *β*_1_ > 0, *β*_2_ < 0, *β*_3_ = 0, and the “turning point” is calculated at *X*_*t*_ = −*β*_1_/2*β*_2_ for models), (4) U-shaped, (5) N-shaped, (6) insignificance (INSIG), and (7) none [31].

### 2.3. Data Analysis

This study used Eviews.6.0 in analyzing the crop planting area, number of livestock breeds, agriculture population, and per capita income of farmers in Ningxia from 1990 to 2008 and Origin 7.5 in making graphics. Data were obtained from Ningxia Statistical Yearbook by Ningxia Bureau of Statistics.

## 3. Results

### 3.1. Evaluation of the Nonpoint Source Pollution Load of the Ningxia Yellow River Irrigation Districts

#### 3.1.1. Pollutant Loads from Agricultural Lands

**Figure 2:**
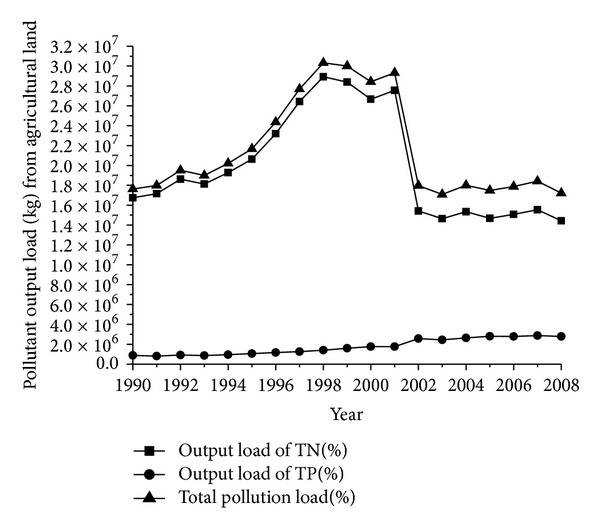
Pollutant output load (kg) from agricultural land in 1990–2008.

The main food crops in the Ningxia Yellow River irrigation region are rice, corn, and wheat. Hence, the study adopted an area-weighted average of these three crops as the pollutant output coefficient of agricultural lands [32]. Different pollutant output coefficients of agricultural land were calculated according to fertilizer usage and fertilizer churn rate from 1990 to 2008. Calculation results are presented in Figure 2.

#### 3.1.2. Pollutant Output Load of Livestock Discharge

**Figure 3:**
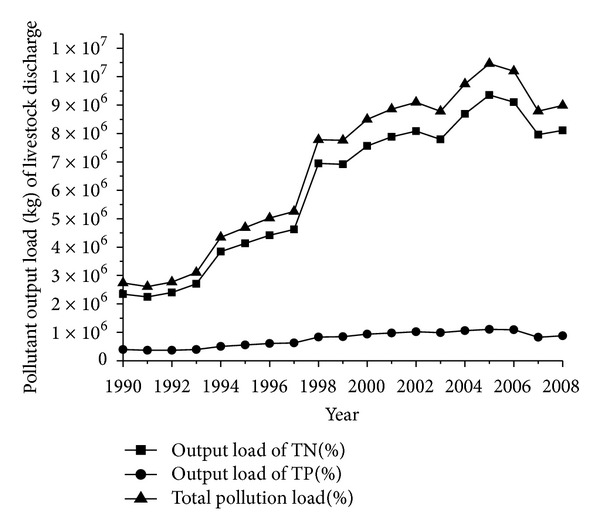
Pollutant output load (kg) of livestock discharge in 1990–2008.

The pollutant output coefficient of livestock discharge represents the total discharge from livestock industries each year (i.e., 365 days). Most of the TN and total phosphorus (TP) pollution came from livestock discharge. Given that the amount of discharge depends on numerous factors, such as livestock species, growth period, forage, and weather, this study adopted reference amount of related livestock to determine the amount of discharge [33, 34]. A pig produces 3.5 kg of feces and 3.5 kg of urine daily; a cow produces 25 kg of feces and 10 kg of urine daily; a sheep produces 2.6 kg of feces and 0.4 kg of urine daily. Normally, the growth period of cattle and sheep is 365 days, whereas that of a pig is 150 days. The nitrogen and phosphorus annual emissions in livestock pollutants are calculated using feces production, output coefficient of nitrogen and phosphorus, and number of livestock by using the following formula: TN and TP annual emissions = individual feces production × feeding period × number of livestock × output coefficient of nitrogen and phosphorus ×10^−3^.  Figure 3 shows the average pollutant content in livestock feces.

#### 3.1.3. Pollution Output Load from the Rural Domestic Sector

**Figure 4:**
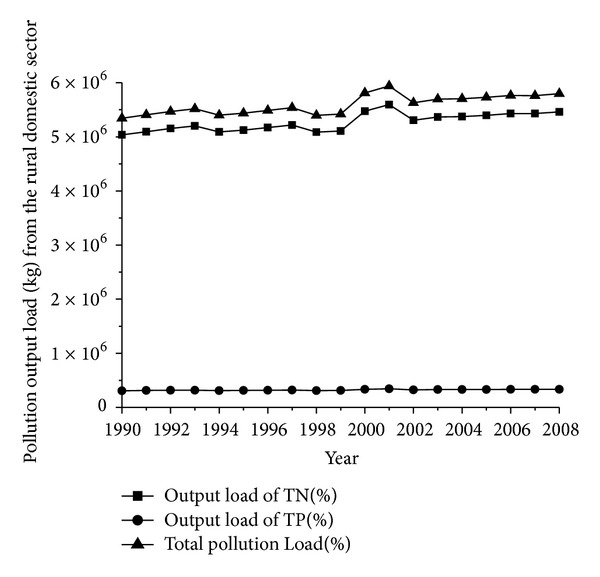
Pollution output load (kg) from the rural domestic sector in 1990–2008.

The distribution of population in rural areas is generally sparse. Rural areas have been polluted to a certain degree because of pollutant discharge. However, current pollutant treatment equipment is still inadequate to remove pollution in these areas. The average nitrogen and phosphorus contents in the per capita domestic sewage per day are 5 and 0.44 g, respectively [35]. Wastage rate is estimated at 100%, given that no unified sewage treatment equipment is available in rural areas. The pollution output load from the rural domestic sector (see Figure 4) can be calculated by using the following formula: amount of annual pollutant discharge = per capita average discharge per day × agricultural population × 365 × levels of pollutants (i.e., TN and TP) × wastage rate.

### 3.2. Empirical Analysis of Pollution Load in the EKC Equation

#### 3.2.1. Testing the Relationship between the Agricultural Pollution Load and the Per Capita Income Growth

Table 1 shows that the result of the quadratic regression is perfect, but the determination coefficient (0.47) is not ideal. Thus, the relationship did not fit the EKC hypothesis. The relationship between the agricultural pollution load and the per capita income has a typical inverted-U EKC feature because the second-order coefficient is negative. The regression equation is as follows:

(4) $$\begin{array}{c} E_{\text{plant}}=-18.4389+7.6465y-0.5115y^{2}. \end{array}$$

First, the derivative of the regression equation was obtained, where the inflection point reflects the per capita income of farmers. The calculation results denote that a per capita income of 1762.49 RMB results in the occurrence of the inflection point of the agricultural industry. In an economic perspective, this result implies that a per capita income below 1762.49 RMB corresponds to gains in the agricultural industry. Consequently, a per capita income equal to or higher than 1762.49 RMB corresponds to losses in the agricultural industry. The per capita income of farmers in the Ningxia Yellow River irrigation districts was nearly 1762.49 RMB in 1997; thus, the planting industry in this region currently experiences a downward trend (see Table 1).

#### 3.2.2. Testing the Relationship between Livestock-Breeding Pollution Load and Per Capita Income Growth

As shown in Table 2, the effects of the quadratic and cubic regressions are perfect; the *t*-test results show that the effect of the quadratic regression is relatively good. The regression equation is expressed as follows:

(5) $$\begin{array}{c} E_{\text{animal}}=-11.2718+4.5599y-0.2524y^{2}. \end{array}$$

The relationship between livestock-breeding pollution load and per capita income presents a typical inverted U-shaped EKC feature because the second-order coefficient is negative. The results of the regression equation were derived, and the turning point of the per capita income was obtained. The results show that the per capita income of farmers was approximately 8386.74 RMB when pollution load reaches its turning point. In terms of economics, this finding indicates that the livestock-breeding pollution load increases with increasing the per capita income of farmers when the latter is below 8386.74 RMB; otherwise, an opposite trend is shown when the income reaches or exceeds 8386.74 RMB. The per capita income of farmers has constantly increased in the Ningxia Yellow River irrigation districts from 1990 to 2008 and has reached 4559.8 RMB in 2008. Thus, pollution load will continue to increase until the income of farmers reaches 8386.76 RMB.

#### 3.2.3. Testing the Relationship between Living-Sewage Pollution Load and Per Capita Income Growth

Table 3 shows the regression analysis of living-sewage pollution load and per capita income growth. As shown in the table, the fitting effects of the quadratic and cubic regressions are imperfect, and the *t*-test of the coefficients is insignificant. Therefore, the relationship between living-sewage pollution load and per capita income growth does not fit the EKC model.

## 4. Discussion

The EKC hypothesis implies that a low total population and slow economic growth rate have been observed before economic growth; thus, the development and utilization of resources have been limited, and the negative influence of technology on the environment has been negligible. During the initial phase of economic growth, the development-resource intensive industry and the polluting technology, combined with population growth, have significantly hastened economic growth, thereby resulting in increased environmental pollution. As the EKC reflected that technological, political, economic conditions and environmental awareness existed at the same time [36], after economic development reached a certain level, the human capital and clean technology intensive industries have played an important role in decreasing environmental degradation. In addition, with the increased awareness of environmental hazards, greater public demands for a healthier and cleaner environment have become popular [13]. People prefer to see the low-income countries turn their attention to protect the environment at earlier stages of development than creating pressure for environmental protection, and they pay more attention to sustainable development. Furthermore, environmental regulations also have played a significant role in environmental protection and the more sustainable policies can create higher levels of development at a lower environmental cost to seek “win-win” policies that yield both economic and environmental gains, such as environmental legislation and market-based incentives, import instead of exploitation of resources to reduce environmental degradation. In a word, at higher levels of development, combining structural change about information-intensive industries and services with increased environmental awareness, adoption of environmental regulations, better technology, and higher environmental expenditures, results in gradual decline of environmental degradation [37].

The EKC hypothesis, which is solely based on the perspective of economics, analyzes the relationship between environment pollution load and economic development. The inverted-U hypothesis summarizes economic growth practices. Economic growth mode and economic formation are different in each region. Therefore, the two factors are not guaranteed to have the same patterns in environmental pollution [38]. Aside from income, many factors such as natural and social factors, environmental policy of a county or region, investment environment, and public consciousness of environmental protection influence the environment [39]. The irreversible ecological threshold is characterized by an inverted U-shaped feature. When this instance occurs, the possibility of solving environmental problems will drop to zero. If the environment pollution reaches a level that is higher than the ecological irreversible threshold, then the destruction of the ecological environment cannot be restored.

The Ningxia Yellow River irrigation area benefits from western development; thus, economic development induces negligible influence to the environment. The environmental awareness of farmers during farming operations constantly improves with increased investments in education. Improving the national economy and the income of farmers results in improved farm facilities and increased utilization of agricultural technology. Moreover, precision fertilization and controlled pesticide use effectively help in the prevention of agricultural pollution. Currently, the demand for meat products is still high, and the breeding industry has become the main source of income in these areas. Therefore, pollution load will increase because of breeding expansion and increased per capita income.

## 5. Conclusions

This study shows that crop cultivation and livestock-breeding pollution load in the Ningxia Yellow River irrigation area fit the EKC hypothesis. Agricultural pollution load and per capita income reached their turning point in 1997. Thus, pollution load gradually decreases as the income increases. However, this finding is not a good indicator for the decrease in total pollution because the above result utilizes an environmental economics point of view. The regression relation of the per capita income of farmers and the livestock-breeding pollutants in the last 19 years shows typical characteristics of EKC; however, livestock-breeding emission is consistently increasing. Much time is needed before the per capita income of farmers reaches 8386.74 RMB, which is the turning point for this factor, because the per capita income of farmers is only 4559.8 RMB in 2008. In addition, with the population growth, demands for resources, such as drinking water, energy use, and traffic volumes, are still increasing with the waste generation increasing in quantity and toxicity which can produce nonpoint resource pollution. Thus, pollution load will continue to increase with increasing income, and this phenomenon will increase in severity after ten years. Thus, it is the priority to ascertain the relationship between the economic growth and environmental quality of Ningxia Yellow River irrigation area for developing environmental management programs and management policy. This study helps us understand the relationship between human activities and pollutant loads to protect environment, especially the aquatic ecosystems. In addition, it is also helpful for identifying the main factors that lead to the agricultural nonpoint source pollution in Ningxia Yellow River irrigation area. Furthermore, our work is important to determine the appropriate mode of economic development of Ningxia Yellow River irrigation area which can be valuable for other researchers. However, further study is needed on the reduction of agricultural pollution and maximization of livestock waste to determine the dispersion of livestock and poultry farms as well as aquaculture farming areas in the Ningxia Yellow River irrigation area. The control of nonpoint source pollution has become an important task of agricultural soil and water environment field, hence, the river basin pollution control should also be paid more attention in the following work, and more work should be done to investigate the comprehensive control measures of agricultural nonpoint source pollution.

## Figures and Tables

**Table 1:** Relationship between agricultural pollution load and per capita income.

Curve type	Constant term	First-order coefficient	Second-order coefficient	Third-order coefficient	*R* ^2^	*F*
Quadratic coefficient	−18.4389 (−2.43)**	7.6465 (3.74)***	−0.5115 (−3.74)***	—	0.47	6.99
Three coefficients	35.0630 (0.26)	−13.8673 (−0.26)	2.3615 (0.33)	−0.1274 (−0.40)	0.47	4.48

*Note*. ***, **, and *, respectively, represent significance levels in 1%, 5%, and 10%. The *t*-test results are written in brackets.

**Table 2:** Relationship between breeding industry pollution load and per capita income.

Curve type	Constant term	First-order coefficient	Second-order coefficient	Third-order coefficient	*R* ^2^	*F*
Quadratic coefficient	−11.2718 (−1.86)*	4.5599 (2.79)**	−0.2524 (−2.31)**	—	0.93	114.48
Three coefficients	172.8145 (1.80)*	−69.4635 (−1.80)*	9.6328 (1.87)*	−0.4385 (−1.92)*	0.95	90.34

*Note*. ***, **, and *, respectively, represent significance levels in 1%, 5%, and 10%. The *t*-test results are written in brackets.

**Table 3:** Relationship between living-sewage pollution load and per capita income.

Curve type	Constant term	First-order coefficient	Second-order coefficient	Third-order coefficient	*R* ^2^	*F*
Quadratic coefficient	9.5409 (10.48)***	−0.2893 (−1.18)	0.0221 (1.35)	—	0.64	14.40
Three coefficients	22.6854 (1.44)	−5.5748 (−0.88)	0.7280 (0.86)	−0.0313 (−0.84)	0.66	9.65

*Note*. ***, **, and *, respectively, represent significance levels in 1%, 5%, and 10%. The *t*-test results are written in brackets.
